# Exploratory Development and Interpretation of an Internally Validated XGBoost-Cox Model Based on Preoperative Inflammation–Nutrition Indices for Overall Survival in Primary Pathological Stage I Rectal Cancer

**DOI:** 10.3390/curroncol33080446

**Published:** 2026-07-25

**Authors:** Ping Huang, Yiqiong Yin, Ziqiang Wang, Zechuan Jin

**Affiliations:** 1 Department of Gastrointestinal and Colorectal Surgery, West China Tianfu Hospital, Sichuan University, Chengdu 610207, China; 2 Colorectal Cancer Center, Department of General Surgery, West China Hospital, Sichuan University, Chengdu 610041, China; 3 Department of General Surgery, State Key Laboratory of Biotherapy and Cancer Center, West China Hospital, Sichuan University, No. 37 Guo Xue Xiang Street, Chengdu 610041, China

**Keywords:** stage I rectal cancer, inflammation–nutrition index, machine learning, XGBoost-Cox, SHAP, decision curve analysis, prognosis

## Abstract

**Background:** Patients with stage I rectal cancer generally have favorable outcomes after curative surgery, but prognosis is not completely homogeneous. This study explored the prognostic association of preoperative inflammation–nutrition indices with overall survival and developed an interpretable internally validated machine learning survival model. **Methods:** We retrospectively included 475 patients with primary pathological stage I rectal adenocarcinoma who underwent curative-intent radical surgery at Sichuan University West China Hospital between 2018 and 2021. Patients downstaged to ypStage I after neoadjuvant therapy or treated by local transanal excision without lymph node dissection were excluded. Candidate predictors included age, sex, carcinoembryonic antigen, and routinely available preoperative inflammation–nutrition indices. LASSO-Cox regression was used for feature selection. Six survival models were developed and evaluated using 1000 bootstrap resamples with out-of-bag internal validation. Model performance was assessed at 60 months using time-dependent AUC, C-index, Brier score, calibration, and decision curve analysis. SHAP analysis was used for model interpretation. **Results:** During a median follow-up of 68 months, 30 deaths occurred. LASSO-Cox regression identified five predictors: age, lymphocyte-to-white blood cell ratio, fibrinogen-to-lymphocyte ratio, albumin-to-alkaline phosphatase ratio, and neutrophil-to-HDL cholesterol ratio. In bootstrap out-of-bag internal validation, XGBoost-Cox achieved the highest, although only marginally higher, discriminative performance among the evaluated models, with a 60-month time-dependent AUC of 0.783, a C-index of 0.774, and a Brier score of 0.0507. The calibration intercept and slope of XGBoost-Cox were 0.519 and 1.070, respectively. SHAP analysis identified age as the most influential predictor, followed by the selected inflammation–nutrition indices. Decision curve analysis suggested potential clinical utility within threshold probabilities from 1% to 20%, although this finding remains exploratory. **Conclusions:** Preoperative inflammation–nutrition indices may contribute to overall survival prognostic stratification in primary pathological stage I rectal cancer. External validation in larger multicenter cohorts is required before clinical application.

## Simple Summary

Patients with stage I rectal cancer usually have favorable outcomes after curative surgery, but survival is not completely homogeneous. Some patients still experience death during follow-up, and conventional staging alone may not fully capture individual differences in prognosis. This study explored whether routinely available preoperative blood-based inflammation and nutrition indices could help stratify overall survival risk in patients with primary pathological stage I rectal cancer. We developed and internally validated several survival prediction models and used SHAP analysis to interpret the final XGBoost-Cox model. Age was the most influential predictor, and four blood-derived indices—LWR, FLR, AAPR, and NHR—also contributed to prediction. However, because only 30 deaths occurred and no external validation was performed, the model should be considered exploratory. Further multicenter studies are needed before this approach can be used in clinical practice.

## 1. Background

Rectal cancer remains a major global health burden and contributes substantially to cancer-related morbidity and mortality worldwide [1,2]. With the increasing use of screening programs, colonoscopy, high-resolution pelvic imaging, and standardized pathological assessment, more patients are diagnosed at an early stage [3]. Stage I rectal cancer is generally defined as pathological T1–T2, N0, M0 rectal adenocarcinoma after curative-intent resection. Although this subgroup usually has favorable long-term outcomes, prognosis is not completely uniform [4,5]. Some patients still experience recurrence, disease progression, or death after surgery. Therefore, conventional TNM staging alone may not fully capture prognostic heterogeneity among patients with early-stage rectal cancer.

Refined risk stratification is clinically meaningful in this setting. Current postoperative management for stage I rectal cancer is generally less intensive than that for locally advanced disease [6,7]. However, patients with higher predicted risk may benefit from individualized follow-up, geriatric or comorbidity assessment, nutritional evaluation, perioperative optimization, and closer monitoring. Identifying additional prognostic markers that are inexpensive, reproducible, and available before surgery may help improve individualized risk assessment.

Tumor progression is influenced not only by tumor-related characteristics but also by host systemic conditions. Inflammation, immune status, nutritional reserve, coagulation activity, liver function, and lipid metabolism are increasingly recognized as components of the host-tumor interaction [8,9]. Peripheral blood-derived indices are attractive because they can be obtained from routine preoperative tests and may reflect systemic biological processes that are not captured by anatomical staging alone [9,10].

Several inflammation-based and nutrition-based prognostic indices have been studied in colorectal cancer and other malignancies. The neutrophil-to-lymphocyte ratio reflects the balance between neutrophil-mediated inflammation and lymphocyte-mediated antitumor immunity [9]. The platelet-to-lymphocyte ratio may capture platelet-related inflammatory and prothrombotic activity [10]. The lymphocyte-to-monocyte ratio reflects adaptive immune competence relative to monocyte/macrophage-associated inflammation [11]. The systemic immune-inflammation index incorporates neutrophils, lymphocytes, and platelets [12]. The prognostic nutritional index combines albumin and lymphocyte count and reflects nutritional and immune status [13]. The albumin-to-alkaline phosphatase ratio has also been investigated as a composite marker of nutritional reserve, systemic inflammation, hepatic function, and metabolic burden [14].

Despite these previous studies, most available evidence has focused on mixed-stage colorectal cancer or locally advanced rectal cancer. Less is known about whether preoperative inflammation–nutrition indices can help stratify overall survival among patients with primary pathological stage I rectal cancer. In addition, many candidate indices share overlapping laboratory components, and their relative contribution may vary across different populations. Therefore, a data-driven feature selection strategy may help identify a parsimonious subset of informative markers in this specific clinical setting.

Traditional Cox proportional hazards regression has been widely used in survival analysis, but conventional linear models may have limited ability to capture nonlinear relationships and complex interactions among predictors. Machine learning-based survival models offer a flexible approach for modeling complex prognostic patterns [15]. However, their clinical implementation is often limited by interpretability concerns. SHapley Additive exPlanations (SHAP) can quantify the contribution of each predictor to model output and improve transparency at both global and individual levels [16].

Therefore, this study aimed to explore the prognostic value of preoperative inflammation–nutrition indices in patients with primary pathological stage I rectal cancer and to develop an interpretable, internally validated machine learning-based survival prediction model for overall survival.

## 2. Methods

### 2.1. Study Population

This retrospective cohort study included patients with pathologically confirmed primary stage I rectal adenocarcinoma who underwent curative-intent radical surgery at Sichuan University West China Hospital between January 2018 and December 2021. Rectal cancer was defined as rectal adenocarcinoma located within 12 cm from the anal verge. Pathological tumor stage was determined according to the 8th edition of the American Joint Committee on Cancer staging system, with reference to the corresponding Union for International Cancer Control TNM 8th edition anatomical classification [17]. Stage I rectal cancer was defined as pathological T1–T2, N0, M0 disease after curative-intent resection.

The study period was selected to ensure adequate follow-up for evaluating 60-month overall survival, as the last follow-up date was 31 March 2026. Including more recent patients would have resulted in insufficient follow-up for 5-year outcome assessment. In addition, staging and treatment practices during this period were relatively consistent at our institution, and electronic medical records and preoperative laboratory data were relatively complete and standardized.

Between January 2018 and December 2021, a total of 5216 patients underwent surgery for rectal cancer at Sichuan University West China Hospital. Among them, 866 patients had postoperative pathological stage I rectal cancer. Patients who underwent local transanal excision without lymph node dissection were excluded because pathological nodal status could not be adequately assessed. Patients who were downstaged to stage I after neoadjuvant therapy were also excluded because ypStage I disease after neoadjuvant treatment may not represent primary untreated stage I disease. After excluding 98 patients who underwent local transanal excision without lymph node dissection and 293 patients who were downstaged to stage I after neoadjuvant therapy, 475 patients were included in the final analysis. These patients accounted for 9.1% of all rectal cancer surgeries and 54.8% of postoperative pathological stage I rectal cancer cases during the study period. The patient selection process is shown in Supplementary Figure S1.

Eligible patients were required to have pathologically confirmed rectal adenocarcinoma, primary pathological stage I disease after curative-intent radical surgery, available preoperative laboratory data, and complete survival follow-up information. Patients were excluded if they had a history of other malignant tumors, received neoadjuvant therapy before surgery, had active infection, hematological disease, autoimmune disease, or severe inflammatory disease before surgery, had incomplete key laboratory or survival data, underwent local transanal excision or other local procedures without lymph node dissection, or were lost to follow-up.

This study was conducted in accordance with the Declaration of Helsinki and was approved by the Ethics Committee of Sichuan University West China Hospital. The requirement for informed consent was waived due to the retrospective nature of the study. Clinical trial registration was not applicable.

### 2.2. Outcome Definition

The primary endpoint was overall survival, defined as the time from surgery to death from any cause or the last follow-up. Patients who were alive at the last follow-up were censored. Follow-up information was obtained from medical records, outpatient visits, telephone interviews, and/or institutional follow-up databases. The last follow-up date was 31 March 2026.

### 2.3. Candidate Predictors and Inflammation–Nutrition-Related Indices

Demographic, clinicopathological, and laboratory variables were collected from the electronic medical record system. Candidate predictors included age, sex, carcinoembryonic antigen, and preoperative inflammation–nutrition-related indices. Laboratory tests were performed within 3 days before surgery. Inflammation–nutrition-related indices were calculated from routine blood count, coagulation, liver function, lipid, and biochemical parameters.

The candidate indices included commonly investigated inflammatory and nutritional markers such as neutrophil-to-lymphocyte ratio (NLR), lymphocyte-to-monocyte ratio (LMR), platelet-to-lymphocyte ratio (PLR), and additional composite indices reflecting immune-inflammatory balance, coagulation, nutritional reserve, liver function, and lipid metabolism. All continuous laboratory variables and derived indices were analyzed as continuous predictors without categorization to avoid information loss and arbitrary threshold selection.

### 2.4. Data Preprocessing

Continuous variables were summarized as median with interquartile range, and categorical variables were summarized as frequencies and percentages. Missing data were handled using complete case analysis. Candidate predictors were standardized when required by specific algorithms. Because no universally accepted normal ranges or clinically validated thresholds exist for composite indices such as LWR, FLR, AAPR, and NHR in stage I rectal cancer, these variables were modeled continuously rather than dichotomized.

### 2.5. Feature Selection Using Least Absolute Shrinkage and Selection Operator Cox (LASSO-Cox) Regression

LASSO-Cox regression was used for feature selection because the primary endpoint was time-to-event overall survival with censored observations [18]. The dataset was not high-dimensional in the strict sense that the number of predictors exceeded the number of patients. However, only 30 deaths occurred during follow-up, and the number of candidate clinical and inflammation–nutrition-related variables was relatively large compared with the number of outcome events. Moreover, several derived indices were correlated because they shared common laboratory components, such as neutrophils, lymphocytes, platelets, albumin, fibrinogen, and lipid parameters. Therefore, LASSO-Cox regression was applied to shrink regression coefficients, reduce multicollinearity, and obtain a parsimonious predictor subset before model development.

All continuous laboratory variables and derived inflammation–nutrition indices were entered into the LASSO-Cox model as continuous predictors without categorization. Continuous predictors were standardized before model fitting. The penalty parameter lambda was selected using 50-fold cross-validation, and the lambda.1se criterion was used to obtain a parsimonious model and reduce overfitting. Variables with nonzero coefficients at the selected lambda were retained for subsequent model development.

### 2.6. Model Development

Using the predictors retained by LASSO-Cox regression, six survival prediction models were developed, including XGBoost-Cox, CoxBoost, Ridge-Cox, ElasticNet-Cox, gradient boosting machine Cox model (GBM-Cox), and random survival forest (RSF).

These models were selected to represent commonly used and methodologically distinct approaches for time-to-event prediction. Ridge-Cox and ElasticNet-Cox were included as penalized Cox regression models and served as regularized linear benchmarks [19]. CoxBoost was included as a boosting-based Cox model designed for survival outcomes [20]. GBM-Cox and XGBoost-Cox were selected to represent gradient boosting survival models capable of capturing nonlinear relationships and potential interactions under a Cox proportional hazards objective [20]. Random survival forest was included as a nonparametric ensemble survival model that can accommodate complex predictor effects without requiring a strict proportional hazards assumption [20].

The same LASSO-selected predictor subset was used across all candidate models. This design was chosen to reduce overfitting risk in the setting of only 30 deaths and to ensure that differences in model performance were mainly attributable to modeling algorithms rather than different input variables.

For model development, conservative hyperparameter settings were used to reduce overfitting because only 30 deaths occurred during follow-up. For Ridge-Cox and ElasticNet-Cox, the penalty parameter lambda was selected using 5-fold cross-validation within the bootstrap training sample, and lambda.1se was used. ElasticNet-Cox used alpha = 0.5. For CoxBoost, the optimal number of boosting steps was selected using 5-fold cross-validation with maxstepno = 100 and penalty = 100. For random survival forest, 500 trees were grown with terminal node size = 10 and mtry = max(1, floor(sqrt(p))), where p was the number of predictors. For GBM-Cox, 300 trees were fitted with interaction depth = 1, shrinkage = 0.01, minimum terminal node size = 10, and bag fraction = 0.8. For XGBoost-Cox, the Cox proportional hazards objective was used with eta = 0.03, max_depth = 1, subsample = 0.8, colsample_bynode = 0.8, lambda = 1, alpha = 0.5, and nrounds = 100. The hyperparameter settings and tuning strategy are summarized in Supplementary Table S2.

### 2.7. Model Performance Evaluation

Model performance was evaluated using 1000 bootstrap resamples with out-of-bag assessment. In each bootstrap iteration, patients were sampled with replacement to form a bootstrap training set, and patients not selected into that bootstrap sample were used as the out-of-bag validation set. Each model was refitted in the bootstrap training set and evaluated in the corresponding out-of-bag sample. Bootstrap iterations were excluded from model-specific performance calculation if the out-of-bag sample contained fewer than 30 patients, fewer than two death events, fewer than two death events before 60 months, or non-informative risk predictions.

Performance was assessed at 60 months using the time-dependent area under the receiver operating characteristic curve, concordance index, and inverse probability of censoring weighted Brier score. The 95% confidence intervals were estimated from the empirical distribution of bootstrap out-of-bag performance measures.

Calibration was assessed using both graphical and quantitative approaches. For each model, 60-month predicted mortality risks were compared with observed outcomes using bootstrap out-of-bag predictions. Calibration intercept and calibration slope were calculated by fitting a weighted logistic calibration model for 60-month mortality using inverse probability of censoring weighting. An intercept close to 0 and a slope close to 1 were considered to indicate better calibration.

Decision curve analysis (DCA) was performed to explore the potential clinical utility of the final model. Considering the low 60-month mortality risk in this stage I rectal cancer cohort, net benefit was calculated across threshold probabilities from 1% to 20% and compared with the treat-all and treat-none strategies. DCA was interpreted as an exploratory assessment of potential clinical usefulness rather than evidence of immediate clinical applicability.

### 2.8. SHAP-Based Model Interpretation

SHAP values quantify the contribution of each predictor to the model-derived mortality risk score. Positive SHAP values indicate that a predictor increases the predicted mortality risk score, whereas negative SHAP values indicate that a predictor decreases the predicted mortality risk score.

Mean absolute SHAP values were calculated to quantify global feature importance. SHAP beeswarm plots were used to visualize the distribution, magnitude, and direction of each predictor’s effect on model output. SHAP dependence plots were further generated to explore the relationship between predictor values and their corresponding SHAP values, thereby assessing potential nonlinear effects.

### 2.9. Statistical Analysis

All statistical analyses were performed using R software version 4.5.3. A two-sided *p* value < 0.05 was considered statistically significant where applicable. LASSO-Cox, Ridge-Cox, and ElasticNet-Cox models were implemented using the glmnet package version 4.1-10. XGBoost-Cox was implemented using the xgboost package version 3.2.1.1 with a Cox proportional hazards objective. CoxBoost was implemented using the CoxBoost package version 1.5.1. Random survival forest was implemented using the randomForestSRC package version 3.6.2. Gradient boosting machine Cox models were implemented using the gbm package version 2.2.3. Time-dependent AUC was calculated using the timeROC package version 0.4.1. Prediction error and censoring-related estimation were supported by survival version 3.8-6 and pec version 2025.06.24 where applicable. SHAP-based interpretation was performed using shapviz version 0.10.3 and custom model interpretation functions. Data manipulation and visualization were performed using dplyr version 1.2.1, tidyr version 1.3.2, ggplot2 version 4.0.2, patchwork version 1.3.2.9000, and related R packages.

This study was reported with reference to the Transparent Reporting of a multivariable prediction model for Individual Prognosis Or Diagnosis statement (TRIPOD) [21]. A completed TRIPOD checklist is provided in Supplementary Table S1.

## 3. Results

### 3.1. Patient Characteristics

A total of 475 patients with primary pathological stage I rectal adenocarcinoma were included in this study. The median age was 62 years, and 258 patients were male. The median follow-up duration was 68 months. During follow-up, 30 patients died.

The baseline demographic, clinicopathological, and laboratory characteristics of the study population are summarized in Table 1. Compared with patients who were alive at the last follow-up, patients who died were older and had higher CEA levels, lower LWR, higher NLR, and lower LMR. The distributions of selected inflammation–nutrition-related indices are also presented in Table 1.

### 3.2. Feature Selection by LASSO-Cox Regression

**Figure 1:**
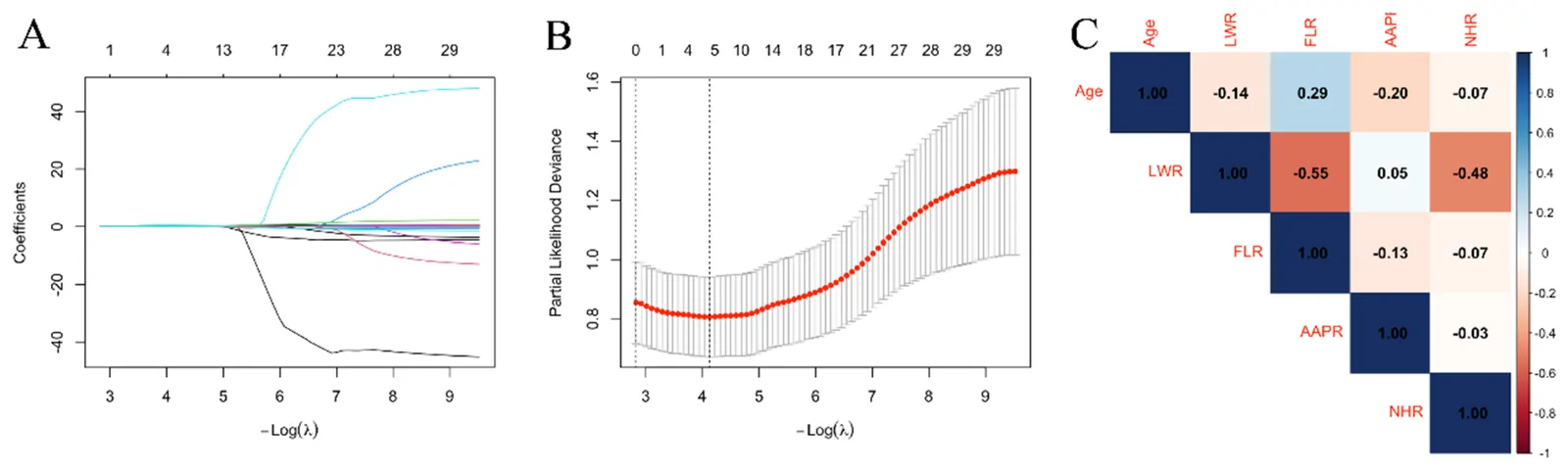
Feature selection using LASSO-Cox regression. (**A**). Coefficient profiles of candidate predictors. (**B**). Cross-validation curve for lambda selection. (**C**). Spearman correlation for all variables. Abbreviations: LWR, lymphocyte-to-white blood cell ratio; FLR, fibrinogen-to-lymphocyte ratio; AAPR, albumin-to-alkaline phosphatase ratio; NHR, neutrophil-to-high-density lipoprotein cholesterol ratio.

LASSO-Cox regression was performed to select prognostic predictors from the candidate variables (Figure 1). CEA was included in the initial candidate predictor set but its coefficient was shrunk to zero at the selected lambda and was therefore not retained. The final retained variables were Age, LWR, FLR, AAPR, and NHR. These variables were used for subsequent machine learning model development.

Pairwise correlations among candidate inflammation–nutrition indices were assessed using Spearman correlation analysis. A correlation heatmap was provided in Figure 1C to visualize potential collinearity. A threshold of |r| ≥ 0.6 was considered to indicate moderate-to-strong correlation. This threshold was used as a pragmatic screening criterion rather than a definitive test of multicollinearity.

### 3.3. Comparison of Survival Machine Learning Models

Six survival prediction models were developed using the five LASSO-selected predictors. Model performance was evaluated using 1000 bootstrap resamples with out-of-bag assessment. The internally validated performance estimates are shown in Table 2.

In bootstrap out-of-bag internal validation, XGBoost-Cox achieved the highest discrimination among the evaluated models, although its advantage over CoxBoost was modest. The 60-month time-dependent AUC of XGBoost-Cox was 0.783, the C-index was 0.774, and the Brier score was 0.0507. CoxBoost showed a similar 60-month time-dependent AUC of 0.782 and a C-index of 0.762. GBM-Cox and random survival forest showed slightly lower discrimination, whereas Ridge-Cox and ElasticNet-Cox showed less stable prediction performance.

Compared with the apparent estimates reported in the original analysis, the bootstrap out-of-bag estimates were more conservative and had wider confidence intervals, reflecting the uncertainty caused by the limited number of deaths. ElasticNet-Cox and Ridge-Cox had fewer valid calibration iterations because these models generated unstable or near-constant 60-month risk predictions in some bootstrap out-of-bag samples.

**Figure 2:**
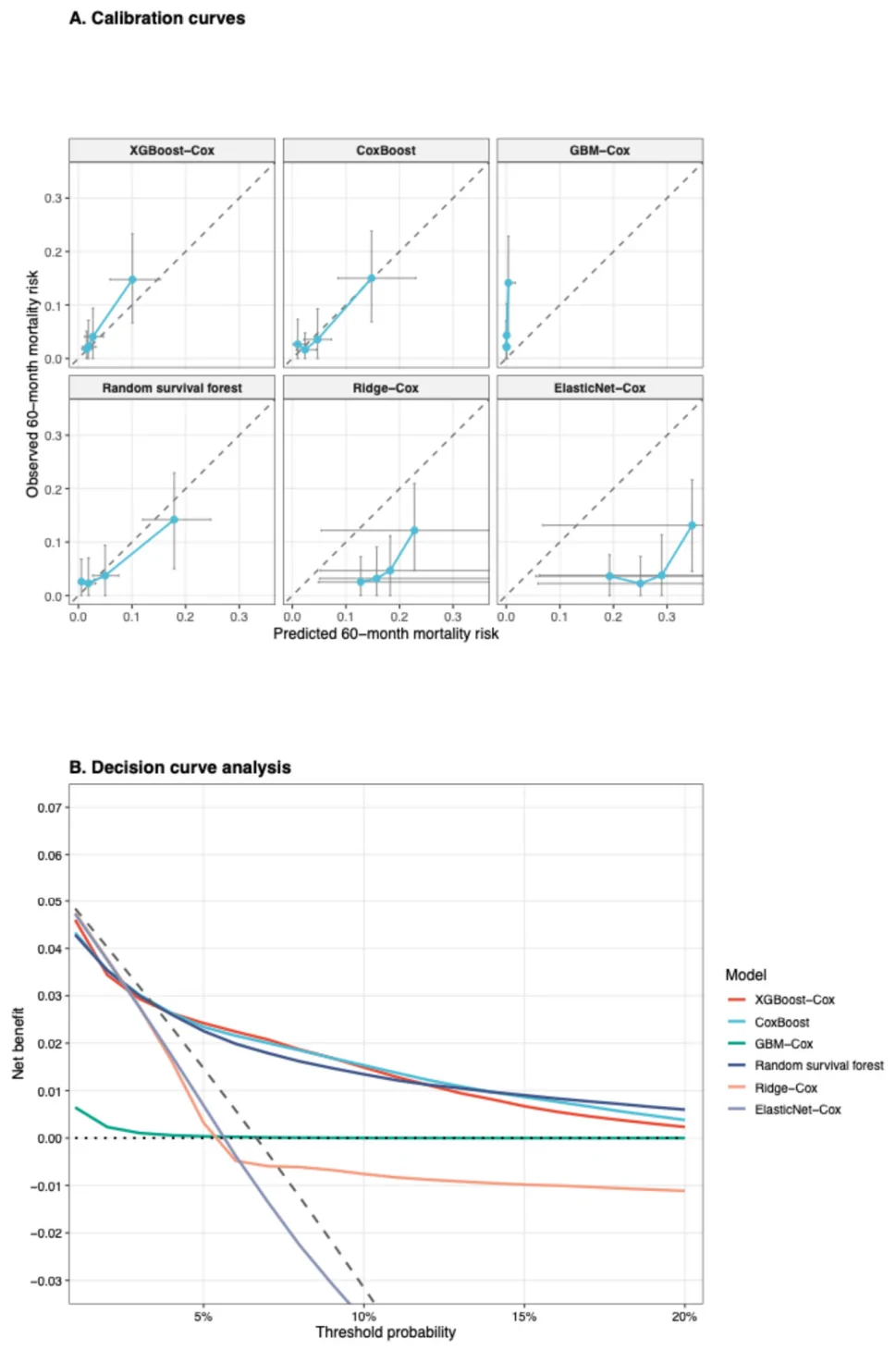
Calibration and decision curve analysis of the survival prediction models. (**A**). Calibration curves comparing predicted and observed 60-month mortality risks. (**B**). Decision curve analysis across threshold probabilities from 1% to 20%. Net benefit was compared with the treat-all (dashed line) and treat-none (dotted line) strategies. Decision curve analysis was interpreted as an exploratory assessment of potential clinical utility.

Calibration was assessed using bootstrap out-of-bag predictions at 60 months (Figure 2A). The XGBoost-Cox model showed a calibration intercept of 0.519 (95% CI: −1.580 to 3.080) and a calibration slope of 1.070 (95% CI: 0.441 to 1.980), suggesting moderate agreement between predicted and observed 60-month mortality risk. However, the calibration estimates had wide confidence intervals, reflecting the small number of death events before 60 months in the out-of-bag samples. Detailed calibration metrics are provided in Supplementary Table S3.

Decision curve analysis (Figure 2B) was performed across threshold probabilities from 1% to 20%, considering the low 60-month mortality risk in this cohort. The XGBoost-Cox model showed potential net benefit across part of this clinically relevant threshold range compared with the treat-all and treat-none strategies. However, because no predefined clinical decision pathway or external validation was available, this finding should be interpreted as exploratory. Therefore, XGBoost-Cox was selected as the final model for SHAP-based interpretation.

### 3.4. SHAP-Based Global Interpretation of the XGBoost-Cox Model

**Figure 3:**
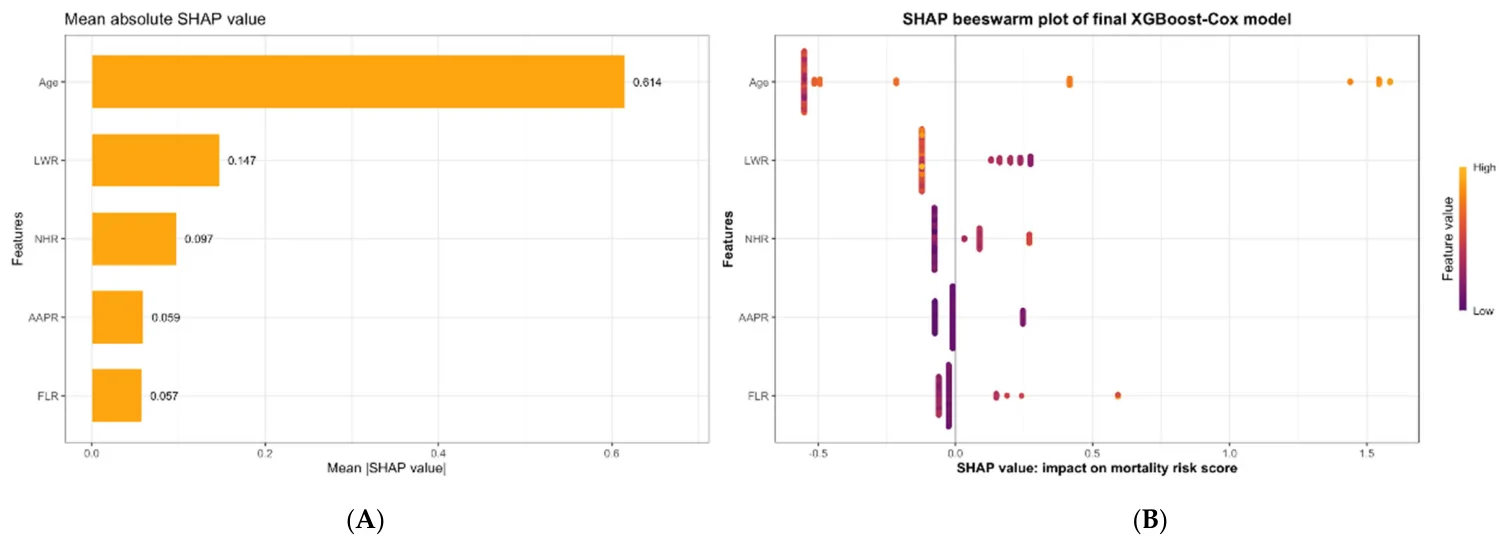
SHAP interpretation of the final XGBoost-Cox model. (**A**). SHAP feature importance plot based on mean absolute SHAP values. (**B**). SHAP beeswarm plot showing the distribution and direction of predictor effects. Abbreviations: LWR, lymphocyte-to-white blood cell ratio; FLR, fibrinogen-to-lymphocyte ratio; AAPR, albumin-to-alkaline phosphatase ratio; NHR, neutrophil-to-high-density lipoprotein cholesterol ratio.

SHAP analysis was performed to interpret the final XGBoost-Cox model (Figure 3). Based on mean absolute SHAP values, Age was the most influential predictor, followed by LWR, NHR, AAPR, and FLR. This ranking indicated that Age had the largest average impact on the model-derived mortality risk score, while inflammation–nutrition-related indices also contributed to prognostic prediction.

In the SHAP beeswarm plot, positive SHAP values indicated an increased model-derived mortality risk score, whereas negative SHAP values indicated a decreased risk score. The color gradient represented the original feature value, with red indicating higher values and blue indicating lower values. The distribution of SHAP values suggested that the magnitude and direction of predictor effects varied across individual patients.

### 3.5. SHAP Dependence Plots

**Figure 4:**
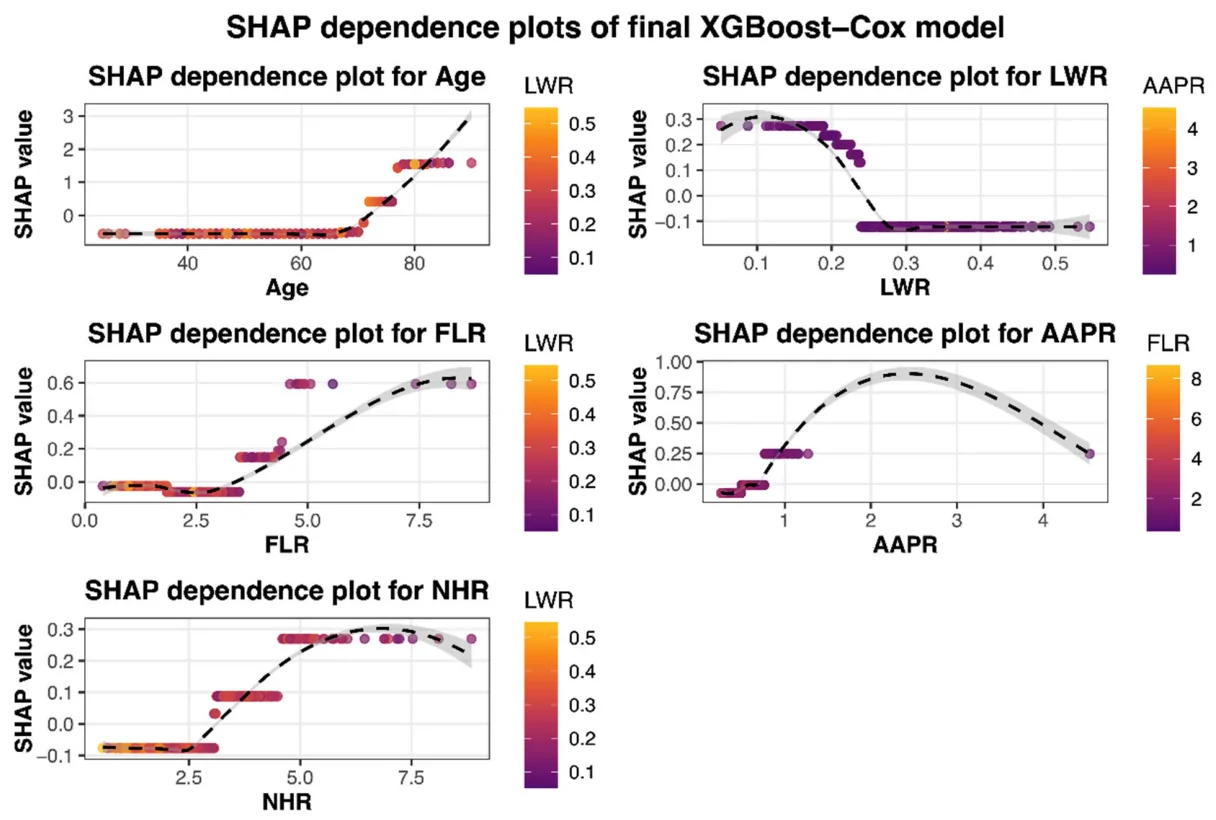
SHAP dependence plots for the five selected predictors. Dependence plots for Age, LWR, FLR, AAPR, and NHR. Abbreviations: LWR, lymphocyte-to-white blood cell ratio; FLR, fibrinogen-to-lymphocyte ratio; AAPR, albumin-to-alkaline phosphatase ratio; NHR, neutrophil-to-high-density lipoprotein cholesterol ratio.

SHAP dependence plots (Figure 4) were generated to further explore the relationship between each predictor value and its corresponding SHAP value. The dependence plots suggested that the selected predictors did not necessarily show strictly linear associations with the model output. Some inflammation–nutrition-related indices showed potential nonlinear effects, indicating that their prognostic contributions may vary across different value ranges. These findings support the use of flexible machine learning-based survival models to capture complex prognostic patterns.

## 4. Discussion

This exploratory retrospective study evaluated the prognostic value of routinely available preoperative inflammation–nutrition indices in patients with primary pathological stage I rectal cancer and developed an interpretable machine learning-based survival model for overall survival. LASSO-Cox regression retained age, LWR, FLR, AAPR, and NHR as predictors. Among the evaluated algorithms, XGBoost-Cox showed the highest, although only modestly higher, internally validated discrimination. SHAP analysis provided insight into the relative contribution of each predictor, and decision curve analysis suggested potential clinical utility within a low-to-moderate threshold probability range. Importantly, because only 30 deaths occurred and no external validation was performed, the model should be interpreted as exploratory and hypothesis-generating rather than ready for clinical implementation.

Although stage I rectal cancer is generally associated with favorable survival, prognosis is not completely homogeneous. Conventional TNM staging classifies patients according to anatomical tumor extent but may not fully reflect host-related prognostic heterogeneity. Established pathological factors, including lymphovascular invasion, perineural invasion, tumor budding, tumor differentiation, margin status, and depth of invasion, are important determinants of prognosis in early rectal cancer [22,23]. However, these variables are obtained after surgical resection and mainly reflect local tumor aggressiveness. In contrast, preoperative inflammation–nutrition indices can be obtained before surgery and may reflect systemic host conditions, including immune function, inflammatory burden, nutritional reserve, coagulation status, hepatic function, and metabolic alterations [13]. Therefore, these indices should be viewed as complementary markers rather than substitutes for conventional pathological assessment.

The selected inflammation–nutrition indices may reflect different but partially overlapping host biological pathways. LWR represents the proportion of lymphocytes within the total white blood cell population. A lower LWR may indicate relative lymphopenia or predominance of non-lymphocyte inflammatory cells, suggesting impaired adaptive antitumor immune surveillance in the context of systemic inflammatory activation [24]. Although NLR is one of the most widely studied inflammatory biomarkers in colorectal cancer, LWR may provide a broader measure of lymphocyte abundance relative to the total leukocyte pool. Because NLR and LWR share overlapping blood cell components, the exclusion of NLR by LASSO-Cox does not imply that NLR lacks prognostic value. Rather, LWR may have provided more stable or non-redundant information in this low-event cohort.

FLR integrates fibrinogen and lymphocyte count. Fibrinogen is both a coagulation factor and an acute-phase reactant [25]. Elevated fibrinogen may facilitate tumor cell adhesion, platelet-tumor interaction, angiogenesis, extracellular matrix remodeling, and immune evasion [25,26]. Conversely, lymphocytes are central to antitumor immunity. Therefore, a higher FLR may reflect a biological state characterized by coagulation-inflammatory activation and weakened adaptive immune competence [27]. This may partly explain why fibrinogen-based indices have been associated with adverse outcomes in colorectal cancer and other malignancies [28].

AAPR combines albumin and alkaline phosphatase. Albumin reflects nutritional reserve, hepatic synthetic function, systemic inflammatory burden, and overall physiological resilience. Lower albumin levels may indicate malnutrition, chronic inflammation, or frailty. Alkaline phosphatase may be related to hepatobiliary function, metabolic stress, bone turnover, and systemic disease burden. A lower AAPR may therefore indicate poorer nutritional status and greater inflammatory or metabolic disturbance [14]. In the present study, AAPR should be interpreted as a composite marker of host reserve rather than a tumor-specific biomarker.

NHR integrates neutrophil-mediated inflammation and HDL-C-related lipid metabolism. Neutrophils can promote cancer progression through cytokine release, reactive oxygen species, neutrophil extracellular traps, angiogenesis, and suppression of cytotoxic immune responses [29]. HDL-C has anti-inflammatory, antioxidant, and immunomodulatory properties and may reflect metabolic health [30]. A higher NHR may therefore indicate heightened neutrophil-driven inflammation combined with reduced HDL-C-associated anti-inflammatory reserve. Although NHR is less established than NLR, PLR, or LMR, it may capture the interaction between systemic inflammation and lipid metabolism, an emerging area of interest in cancer prognosis.

These selected indices likely reflect both distinct and overlapping mechanisms. LWR primarily reflects immune-inflammatory balance, FLR links coagulation and immune status, AAPR reflects nutritional and hepatic-metabolic reserve, and NHR integrates inflammation and lipid metabolism. However, systemic inflammation may simultaneously influence lymphocyte count, neutrophil count, fibrinogen, albumin, lipid metabolism, and liver enzymes. Therefore, the model should be interpreted as capturing a composite host-response phenotype rather than isolated causal pathways.

Previous studies have reported associations between inflammation-based or nutrition-based indices and outcomes in colorectal cancer. NLR, PLR, LMR, SII, PNI, AAPR, and fibrinogen-based markers have been investigated in different colorectal cancer populations, including mixed-stage colorectal cancer, colon cancer, rectal cancer, and patients receiving neoadjuvant or adjuvant therapy [14,31,32]. Many of these studies suggested that systemic inflammatory activation, lymphopenia, hypoalbuminemia, hypercoagulability, and impaired nutritional status are associated with worse prognosis. However, direct comparison with our findings is difficult because most previous studies included heterogeneous stages, different endpoints, variable treatment strategies, and different biomarker cut-offs. Our study differs by focusing specifically on primary pathological stage I rectal cancer and by modeling the selected indices as continuous variables in an interpretable survival machine learning framework.

Age was identified as the most influential predictor in the final model. This finding should be interpreted cautiously because the endpoint was overall survival, which includes death from any cause. Older age is a well-established risk factor for all-cause mortality irrespective of rectal cancer [33]. Older patients may have reduced physiological reserve, more comorbidities, frailty, impaired immune function, and lower tolerance to postoperative complications or subsequent treatments [34]. Therefore, the contribution of age in this model may reflect not only cancer-related prognosis but also competing risks from comorbidities and non-cancer death. Future studies using cancer-specific survival, disease-free survival, recurrence-free survival, or competing-risk methods are needed to clarify whether these predictors are specifically associated with tumor-related outcomes.

XGBoost-Cox achieved the highest discrimination among the candidate models, although its advantage over CoxBoost was modest in bootstrap out-of-bag internal validation. Compared with conventional penalized Cox models, XGBoost-Cox may capture nonlinear relationships and interactions among predictors. SHAP dependence plots also suggested that the effects of selected predictors on the model-derived mortality risk score were not necessarily linear. Nevertheless, because the number of outcome events was limited, algorithm ranking should be interpreted cautiously. The apparent superiority of one flexible algorithm over another may be unstable in small-event datasets.

A major challenge in applying machine learning models to clinical medicine is limited interpretability. To address this issue, we used SHAP analysis to explain the final XGBoost-Cox model. SHAP values quantified the contribution of each predictor to the model output and allowed visualization of both global feature importance and predictor effects [16]. However, SHAP analysis explains model predictions and does not establish causal relationships. In addition, SHAP patterns may be unstable when the model is trained on a small number of outcome events.

Clinical usefulness also requires cautious interpretation. Decision curve analysis suggested potential net benefit across part of the 1% to 20% threshold probability range. However, no predefined clinical decision pathway was available in this retrospective study. Therefore, DCA should be interpreted as exploratory. If externally validated, a high predicted all-cause mortality risk might support closer follow-up, geriatric or comorbidity assessment, nutritional evaluation, and optimization of systemic inflammatory or metabolic conditions. However, the model should not currently be used to determine adjuvant treatment, imaging frequency, or surveillance intensity in routine practice.

The lack of clinically interpretable cut-offs is another practical limitation. In this study, LWR, FLR, AAPR, and NHR were modeled as continuous predictors to avoid information loss and arbitrary dichotomization. There are no universally accepted normal ranges or clinically validated thresholds for these composite indices in stage I rectal cancer. Therefore, we did not define cut-offs for clinical decision-making. Future studies should identify reproducible thresholds and evaluate whether threshold-based risk groups can guide surveillance, nutritional intervention, supportive care, or perioperative optimization.

This study has several strengths. First, it focused on primary pathological stage I rectal cancer, a subgroup generally considered to have favorable prognosis but still showing clinically meaningful heterogeneity. Second, the model was based on routinely available preoperative clinical and laboratory indicators. Third, multiple survival models were compared using the same LASSO-selected predictor subset. Fourth, bootstrap out-of-bag internal validation was used to provide more conservative performance estimates. Finally, SHAP analysis was used to improve model interpretability.

Several limitations should be acknowledged. First, this was a retrospective single-center study, and selection bias could not be completely avoided. Second, only 30 deaths occurred during follow-up, which is the most important methodological limitation. Although we used LASSO-based feature selection, conservative model complexity settings, and bootstrap out-of-bag internal validation, the small number of events may still lead to unstable feature selection, overfitting, wide uncertainty intervals, limited calibration reliability, and unstable algorithm ranking. Therefore, the model should be considered exploratory and hypothesis-generating. Third, the model was evaluated using internal validation only. External validation in independent multicenter cohorts is essential before clinical application. Fourth, the endpoint was overall survival rather than cancer-specific survival or disease-free survival. In stage I rectal cancer, non-cancer mortality is clinically relevant, especially among older patients. Therefore, the model may primarily predict all-cause mortality rather than rectal cancer-specific prognosis. Fifth, detailed pathological variables such as lymphovascular invasion, perineural invasion, tumor budding, tumor differentiation, margin status, and depth of invasion were not consistently available. Therefore, we could not determine whether preoperative inflammation–nutrition indices provide incremental prognostic value beyond established pathological risk factors. Sixth, inflammation–nutrition indices may be influenced by infection, chronic inflammatory disease, metabolic disorders, liver function, medication use, BMI, nutritional interventions, comorbidities, frailty, and other unmeasured confounders. Although patients with active infection, hematological disease, autoimmune disease, or severe inflammatory disease were excluded, detailed information on BMI, comorbidity burden, sarcopenia, medication use, and nutritional interventions was not consistently available. Finally, the proposed model should be compared with previously published prognostic models in future studies. Many existing models for rectal or colorectal cancer incorporate clinicopathological variables, radiological features, molecular markers, treatment factors, or mixed-stage cohorts. Our model used only age and preoperative blood-derived indices in primary pathological stage I rectal cancer. Direct performance comparison is difficult because of differences in populations, endpoints, follow-up duration, event rates, and validation strategies. Future studies should incorporate conventional pathological, radiological, molecular, treatment-related, and host-related variables to develop more comprehensive and externally validated prognostic models.

## 5. Conclusions

This exploratory study suggests that routinely available preoperative inflammation–nutrition indices may contribute to overall survival risk stratification in patients with stage I rectal cancer. An XGBoost-Cox model based on Age, LWR, FLR, AAPR, and NHR showed promising performance in internal evaluation and was interpretable using SHAP. However, due to the limited number of deaths, lack of detailed pathological variables, and absence of external validation, these findings should be interpreted cautiously. Further multicenter studies incorporating conventional pathological, radiological, molecular, treatment-related, and host-related variables are needed before clinical application.

## Figures and Tables

**Table 1:** Baseline characteristics of patients with primary pathological stage I rectal cancer according to survival status.

Variable	Alive, *n* = 445	Dead, *n* = 30	*p* Value
Follow-up time, months (median [IQR])	68.00 [63.00, 77.00]	42.50 [28.50, 49.75]	<0.001
Sex, Male (%)	237 (53.3)	21 (70.0)	0.111
Age (median [IQR])	61.00 [51.00, 69.00]	77.00 [67.50, 79.00]	<0.001
CEA (median [IQR])	2.30 [1.40, 3.63]	3.07 [2.44, 6.96]	0.004
LWR (median [IQR])	0.30 [0.25, 0.35]	0.23 [0.18, 0.32]	0.005
FLR (median [IQR])	1.72 [1.35, 2.29]	1.81 [1.48, 3.20]	0.166
AAPR (median [IQR])	0.57 [0.48, 0.69]	0.56 [0.50, 0.74]	0.593
NHR (median [IQR])	2.45 [1.73, 3.36]	3.10 [2.05, 3.95]	0.052
NLR (median [IQR])	1.98 [1.56, 2.68]	3.03 [1.82, 4.20]	0.009
LMR (median [IQR])	5.17 [3.95, 6.91]	4.37 [3.51, 5.74]	0.032
PLR (median [IQR])	102.01 [80.11, 132.32]	113.28 [76.27, 137.94]	0.535

Continuous variables are presented as median [interquartile range], and categorical variables are presented as *n* (%). Abbreviations: CEA, carcinoembryonic antigen; LWR, lymphocyte-to-white-blood-cell ratio; FLR, fibrinogen-to-lymphocyte ratio; AAPR, albumin-to-alkaline phosphatase ratio; NHR, neutrophil-to-HDL cholesterol ratio; NLR, neutrophil-to-lymphocyte ratio; LMR, lymphocyte-to-monocyte ratio; PLR, platelet-to-lymphocyte ratio.

**Table 2:** Bootstrap out-of-bag internal validation performance of six survival prediction models at 60 months.

Model	Time-Dependent AUC, 95% CI	C-Index, 95% CI	Brier Score, 95% CI	Valid Bootstrap Iterations
XGBoost-Cox	0.783 (0.627–0.915)	0.774 (0.642–0.902)	0.0507 (0.0280–0.0774)	1000
CoxBoost	0.782 (0.629–0.923)	0.762 (0.615–0.895)	0.0528 (0.0308–0.0786)	998
GBM-Cox	0.770 (0.605–0.913)	0.762 (0.615–0.896)	0.0565 (0.0291–0.0865)	1000
Random survival forest	0.748 (0.591–0.901)	0.745 (0.602–0.890)	0.0506 (0.0320–0.0734)	1000
Ridge-Cox	0.743 (0.583–0.887)	0.737 (0.603–0.873)	0.0689 (0.0320–0.2349)	1000
ElasticNet-Cox	0.734 (0.551–0.882)	0.707 (0.550–0.850)	0.1549 (0.0417–0.8063)	341

Values were estimated using 1000 bootstrap resamples with out-of-bag evaluation. Confidence intervals were derived from the empirical distribution of bootstrap out-of-bag performance estimates. ElasticNet-Cox had fewer valid bootstrap iterations because the model failed to generate stable non-constant risk predictions in a substantial proportion of out-of-bag evaluations.

## Data Availability

The datasets used and/or analyzed during the current study are available from the corresponding author on reasonable request.

## Supplementary Materials

The following supporting information can be downloaded at: https://www.mdpi.com/article/10.3390/curroncol33080446/s1, Figure S1: Flow diagram of patient selection; Table S1: TRIPOD checklist; Table S2: Hyperparameter settings and tuning strategy for candidate survival models; Table S3: Bootstrap out-of-bag corrected calibration performance of candidate survival models at 60 months.

## Abbreviations

AUC	area under the receiver operating characteristic curve
C-index	concordance index
DCA	decision curve analysis
GBM	gradient boosting machine
HDL-C	high-density lipoprotein cholesterol
LASSO	least absolute shrinkage and selection operator
OS	overall survival
RSF	random survival forest
SHAP	SHapley Additive exPlanations
XGBoost	extreme gradient boosting
CEA	Carcinoembryonic antigen
NLR	Neutrophil-to-lymphocyte ratio
LMR	Lymphocyte-to-monocyte ratio
PLR	Platelet-to-lymphocyte ratio
LWR	Lymphocyte-to-white-blood-cell ratio
NMR	Neutrophil-to-monocyte ratio
PMR	Platelet-to-monocyte ratio
NPR	Neutrophil-to-platelet ratio
LPR	Lymphocyte-to-platelet ratio
NAR	Neutrophil-to-albumin ratio
MAR	Monocyte-to-albumin ratio
FAR	Fibrinogen-to-albumin ratio
PFR	Platelet-to-fibrinogen ratio
FLR	Fibrinogen-to-lymphocyte ratio
AAPR	Albumin-to-alkaline phosphatase ratio
LAR	Lactate dehydrogenase-to-albumin ratio
LLR	Lactate dehydrogenase-to-lymphocyte ratio
GPR	Gamma-glutamyl transferase-to-platelet ratio
GLR	Gamma-glutamyl transferase-to-lymphocyte ratio
ALRI	AST-to-lymphocyte ratio index
ANRI	AST-to-neutrophil ratio index
MHR	Monocyte-to-HDL cholesterol ratio
NHR	Neutrophil-to-HDL cholesterol ratio
LHR	Lymphocyte-to-HDL cholesterol ratio
HPR	Hemoglobin-to-platelet ratio
RAR	Red cell distribution width-to-albumin ratio
