# Supplementary material for: Exploratory Development and Interpretation of an Internally Validated XGBoost-Cox Model Based on Preoperative Inflammation–Nutrition Indices for Overall Survival in Primary Pathological Stage I Rectal Cancer
Source: Curr Oncol. 2026 Jul 25;33(8):446. doi: 10.3390/curroncol33080446 (PMC13510550; doi:10.3390/curroncol33080446)
Supplement: Supplementary file 1 [file curroncol-33-00446-s001.zip › Supplementary Table S2.pdf]

Supplementary Table S2. Hyperparameter settings and tuning strategy for candidate survival models.

Model	Package / function	Hyperparameter setting or tuning strategy
Ridge-Cox	glmnet / cv.glmnet	alpha = 0; lambda selected by 5-fold cross-validation; lambda.1se was used; predictors were standardized.
ElasticNet-Cox	glmnet / cv.glmnet	alpha = 0.5; lambda selected by 5-fold cross-validation; lambda.1se was used; predictors were standardized.
CoxBoost	CoxBoost / cv.CoxBoost	maxstepno = 100; K = 5; type = "verweij"; penalty = 100; optimal boosting step selected by cross-validation.
Random survival forest	randomForestSRC / rfsrc	ntree = 500; nodesize = 10; mtry = max(1, floor(sqrt(number of predictors))); importance = FALSE; block.size = 1.
GBM-Cox	gbm / gbm	distribution = "coxph"; n.trees = 300; interaction.depth = 1; shrinkage = 0.01; n.minobsinnode = 10; bag.fraction = 0.8; train.fraction = 1.0.
XGBoost-Cox	xgboost / xgb.train	objective = "survival:cox"; eval_metric = "cox-nloglik"; eta = 0.03; max_depth = 1; subsample = 0.8; colsample_bynode = 0.8; lambda = 1; alpha = 0.5; nrounds = 100.

The same five LASSO-selected predictors were used across all candidate models. Conservative model complexity settings were adopted because only 30 deaths occurred during follow-up. In the bootstrap out-of-bag validation procedure, each model was refitted within the bootstrap training sample and evaluated in the corresponding out-of-bag sample.
