# Supplementary material for: Exploratory Development and Interpretation of an Internally Validated XGBoost-Cox Model Based on Preoperative Inflammation–Nutrition Indices for Overall Survival in Primary Pathological Stage I Rectal Cancer
Source: Curr Oncol. 2026 Jul 25;33(8):446. doi: 10.3390/curroncol33080446 (PMC13510550; doi:10.3390/curroncol33080446)
Supplement: Supplementary file 1 [file curroncol-33-00446-s001.zip › Supplementary Table S3.pdf]

Supplementary Table S3. Bootstrap out-of-bag corrected calibration performance of candidate survival models at 60 months.

Model	Calibration intercept, mean (95% CI)	Calibration slope, mean (95% CI)	Valid bootstrap iterations	Median number of events before 60 months
CoxBoost	-0.441 (-2.770 to 3.700)	0.856 (0.101 to 2.500)	998	10
ElasticNet-Cox	5.750 (-3.960 to 42.700)	4.490 (0.094 to 19.500)	341	10
GBM-Cox	4.100 (-0.150 to 9.390)	0.983 (0.395 to 1.830)	1000	10
Random survival forest	-0.836 (-2.650 to 1.380)	0.719 (0.132 to 1.780)	1000	10
Ridge-Cox	29.800 (-3.560 to 229.000)	12.600 (0.155 to 83.400)	317	10
XGBoost-Cox	0.519 (-1.580 to 3.080)	1.070 (0.441 to 1.980)	1000	10

Calibration metrics were estimated using bootstrap out-of-bag predictions at 60 months. Calibration intercept and slope were calculated by fitting a weighted logistic calibration model for 60-month mortality using inverse probability of censoring weighting. A calibration intercept close to 0 and a calibration slope close to 1 indicate better agreement between predicted and observed mortality risk. The wide confidence intervals reflect the limited number of 60-month death events in the out-of-bag samples.
